# Supplementary material for: Discovery and application of insertion-deletion (INDEL) polymorphisms for QTL mapping of early life-history traits in Atlantic salmon
Source: BMC Genomics. 2010 Mar 8;11:156. doi: 10.1186/1471-2164-11-156 (PMC2838853; doi:10.1186/1471-2164-11-156)
Supplement: Additional file 2 — Information on developed 76 locus single-run INDEL panel in Atlantic salmon. Information on fluorescence labeling, primer concentrations, PCR pooling and links to alignments, INDEL motifs and GENESCAN (Burge and Karlin 1997) predictions of genes/exons are available in html format. [file 1471-2164-11-156-S2.ZIP › Additionalfile2/table12783.html]

Contig Alignment

```
Base	A B C D E F G H I J K   
1	G . G . . . . . . . .    
2	G . G . . . . . . . .    
3	G . G . . . . . . . .    
4	G . G . . . . . . . .    
5	G . G . . . . . . . .    
6	T T T . . . . . . . .    
7	G G G . . . . . . . .    
8	G G G . . . . . . . .    
9	T T T . . . . . . . .    
10	C C C . C . . . . . .    
11	G G G . G . . . . . .    
12	A A A . A . . . . . .    
13	G G G . G . . . . . .    
14	T T T . T . . . . . .    
15	C C C . C . . . . . .    
16	T T T . T . . . . . .    
17	G G G . G . . . . . .    
18	A A A . A . . . . . .    
19	G G G . G . . . . . .    
20	T T T . T . . . . . .    
21	T T T . T . . . . . .    
22	T T T . T . . . . . .    
23	T T T . T . . . . . .    
24	A A A . A . . . . . .    
25	T T T . T . . . . . .    
26	A A A . A . . . . . .    
27	C C C . C . . . . . .    
28	C C C . C . . . . . .    
29	A A A A A . . . . . .    
30	C C C C C . . . . . .    
31	A A A A A . . . . . .    
32	C C C C C . . . . . .    
33	G G G G G . . . . . .    
34	G G G G G . . . . . .    
35	C C C C C . . . . . .    
36	G G G G G . . . . . .    
37	T T T T T . . . . . .    
38	G G G G G . . . . . .    
39	G G G G G . . . . . .    
40	A A A A A . . . . . .    
41	A A A A A . . . . . .    
42	A A A A A . . . . . .    
43	C C C C C . . . . . .    
44	G G G G G . . . . . .    
45	A A A A A . . . . . .    
46	G G G G G . . . . . .    
47	C C C C C . . . . . .    
48	T T T T T . . . . . .    
49	C C C C C . . . . . .    
50	G G G G G . . . . . .    
51	C T T T T . . . . . .    
52	G G G G G . . . . . .    
53	G G G G G . . . . . .    
54	A A A A A . . . . . .    
55	A A A A A . . . . . .    
56	C C C C C . . . . . .    
57	A A A A A . . . . . .    
58	T T T T T . . . . . .    
59	T T T T T . . . . . .    
60	T T T T T . . . . . .    
61	A A A A A . . . . . .    
62	G G G G G . . . . . .    
63	G G G G G . . . . . .    
64	A A A A A . . . . . .    
65	T T T T T . . . . . .    
66	T T T T T . . . . . .    
67	A A A A A . . . . . .    
68	A A A A A . . . . . .    
69	A A A A A . . . . . .    
70	A A A A A . . . . . .    
71	G G G G G . . . . . .    
72	T T T T T . . . . . .    
73	C C C C C . . . . . .    
74	A A A A A . . . . . .    
75	C C C C C . . . . . .    
76	A A A A A . . . . . .    
77	G G G G G . . . . . .    
78	T T T T T . . . . . .    
79	T T T T T . . . . . .    
80	T T T T T . . . . . .    
81	A A A A A . . . . . .    
82	C C C C C . . . . . .    
83	T T T T T . . . . . .    
84	G G G G G . . . . . .    
85	A A A A A . . . . . .    
86	A A A A A . . . . . .    
87	A A A A A . . . . . .    
88	A A A A A . . . . . .    
89	A A A A A . . . . . .    
90	C C C C C . . . . . .    
91	A A A A A . . . . . .    
92	A A A A A . . . . . .    
93	A A A A A . . . . . .    
94	C C C C C . . . . . .    
95	C C C C C . . . . . .    
96	T T T T T . . . . . .    
97	A A A A A A . . . . .    
98	T T T T T T . . . . .    
99	T T T T T T . . . . .    
100	G G G G G G . . . . .    
101	C C C C C C . . . . .    
102	A A A A A A . . . . .    
103	G G G G G G . . . . .    
104	T T T T T T . . . . .    
105	C C C C C C . . . . .    
106	A A A A A A . . . . .    
107	C C C C C C . . . . .    
108	T T T T T T . . . . .    
109	C C C C C C . . . . .    
110	A A A A A A . . . . .    
111	G G G G G G . . . . .    
112	A A A A A A . . . . .    
113	A A A A A A . . . . .    
114	G G G G G G . . . . .    
115	A A A A A A . . . . .    
116	C C C C C C . . . . .    
117	G G G G G G . . . . .    
118	G G G G G G . . . . .    
119	A A A A A A . . . . .    
120	C C C C C C . . . . .    
121	A A A A A A . . . . .    
122	T T T T T T . . . . .    
123	A A A A A A . . . . .    
124	T T T T T T . . . . .    
125	A A A A A A . . . . .    
126	G G G G G G . . . . .    
127	T T T T T T . . . . .    
128	T T T T T T . . . . .    
129	T T T T T T . . . . .    
130	A A A A A A . . . . .    
131	G G G G G G . . . . .    
132	T T T T T T . . . . .    
133	G G G G G G . . . . .    
134	T T T T T T . . . . .    
135	C C C C C C . . . . .    
136	A A A A A A . . . . .    
137	A A A A A A . . . . .    
138	C C C C C C . . . . .    
139	A A A A A A . . . . .    
140	G G G G G G . . . . .    
141	T T T T T T . . . . .    
142	A A A A A A . . . . .    
143	A A A A A A . . . . .    
144	C C C C C C . . . . .    
145	G G G G G G . . . . .    
146	T T T T T T . . . . .    
147	C C C C C C . . . . .    
148	A A A A A A . . . . .    
149	C C C C C C . . . . .    
150	A A A A A A . . . . .    
151	A A A A A A . . . . .    
152	G G G G G G . . . . .    
153	A A A A A A . . . . .    
154	A A A A A A . . . . .    
155	T T T T T T . . . . .    
156	C C C C C C . . . . .    
157	T T T T T T . . . . .    
158	C C C C C C . . . . .    
159	C C C C C C . . . . .    
160	A A A A A A . . . . .    
161	A A A A A A . . . . .    
162	T T T T T T . . . . .    
163	T T T T T T . . . . .    
164	G G G G G G . . . . .    
165	A A A A A A . . . . .    
166	A A A A A A . . . . .    
167	A A A A A A . . . . .    
168	T T T T T T . . . . .    
169	G G G G G G . . . . .    
170	G G G G G G . . . . .    
171	C C C C C C . . . . .    
172	A A G A A A . . . . .    
173	A A A A A A . . . . .    
174	A A A A A A . . . . .    
175	T T T T T T . . . . .    
176	G G G G G G . . . . .    
177	A A A A A A . . . . .    
178	C C C C C C . . . . .    
179	A A A A A A . . . . .    
180	A A A A A A . . . . .    
181	C C C C C C . . . . .    
182	G G G G G G . . . . .    
183	C C C C C C . . . . .    
184	G G G G G G . . . . .    
185	A A A A A A . . . . .    
186	A A A A A A . . . . .    
187	A A A A A A . . . . .    
188	C C C C C C . . . . .    
189	T T T T T T . . . . .    
190	C C C C C C . . . . .    
191	T T T T T T . . . . .    
192	C C C C C C . . . . .    
193	C C C C C C . . . . .    
194	A A A A A A . . . . .    
195	A A A A A A . . . . .    
196	A A A A A A . . . . .    
197	A A A A A A . . . . .    
198	A A A A A A . . . . .    
199	C C C C C C . . . . .    
200	C C C C C C . . . . .    
201	T T T T T T . . . . .    
202	C C C C C C . . . . .    
203	T T T T T T . . . . .    
204	T T T T T T . . . . .    
205	G G G G G G . . . . .    
206	C C C C C C . . . . .    
207	G G G G G G . . . . .    
208	A A A A A A . A . . .    
209	A A A A A A . A . . .    
210	T T T T T T . T . . .    
211	G G G G G G . G . . .    
212	A A A A A A . A . . .    
213	A A A A A A . A . . .    
214	G G G G G G . G . . .    
215	T T T T T T . T . . .    
216	T T T T T T . T . . .    
217	C C C C C C C C . . .    
218	A A A A A A A A . . .    
219	T T T T T T T T . . .    
220	G G G G G G G G . . .    
221	C C C C C C C C . . .    
222	A A A A A A A A . . .    
223	A A A A A A A A . . .    
224	A A A A A A A A . . .    
225	G G G G G G G G . . .    
226	A A A A A A A A . . .    
227	G G G G G G G G . . .    
228	G G G G G G G G . . .    
229	C C C C C C C C . . .    
230	C C C C C C C C . . .    
231	T T T T T T T T . . .    
232	G G G G G G G G . . .    
233	G G G G G G G G . . .    
234	A A A A A A A A . . .    
235	T T T T T T T T . . .    
236	G G G G G G G G . . .    
237	C C C C C C C C . . .    
238	A A A A A A A A . . .    
239	G G G G G G G G . . .    
240	A A A A A A A A . . .    
241	T T T T T T T T . . .    
242	A A A A A A A A . . .    
243	C C C C C C C C . . .    
244	A A A A A A A A . . .    
245	A A A A A A A A . . .    
246	A A A A A A A A . . .    
247	G G G G G G G G . . .    
248	A G A A A G A A . . .    
249	A A A A A A A A . . .    
250	G G G G G G G G . . .    
251	C C C C C C C C . . .    
252	A A A A A A A A . . .    
253	A A A A A A A A . . .    
254	C C C C C C C C . . .    
255	T T T T T T T T . . .    
256	G G G G G G G G . . .    
257	G G G G G G G G . . .    
258	A A A A A A A A . . .    
259	A A A A A A A A . . .    
260	G G G G G G G G . . .    
261	A A A A A A A A . . .    
262	A A A A A A A A . . .    
263	G G G G G G G G . . .    
264	A A A G A A A A . . .    
265	G G G G G G G G . . .    
266	G G G G G G G G . . .    
267	A A A A A A A A . . .    
268	G G G G G G G G . . .    
269	A A A A A A A A . . .    
270	G G G G G G G G . . .    
271	G G G G G G G G . . .    
272	A A A A A A A A . . .    
273	G G G G G G G G . . .    
274	G G G G G G G G . . .    
275	A A A A A A A A . . .    
276	T T T T T T T T . . .    
277	C C C C C C C C . . .    
278	A A A A A A A A . . .    
279	T T T T T T T T . . .    
280	C C C C C C C C . . .    
281	A A A A A A A A . . .    
282	G G G G G G G G . . .    
283	T T T T T T T T . . .    
284	G G G G G G G G . . .    
285	A A A A A A A A . . .    
286	T T T T T T T T . . .    
287	G G G G G G G G . . .    
288	A A A A A A A A . . .    
289	G G G G G G G G . . .    
290	C C C C C C C C . . .    
291	A A A A A A A A . . .    
292	T T T T T T T T . . .    
293	T T T T T T T T . . .    
294	G G G G G G G G . . .    
295	G G G G G G G G . . .    
296	T T T T T T T T . . .    
297	A A A A A A A A . . .    
298	T T T T T T T T . . .    
299	C C C C C C C C . . .    
300	T T T T T T T T . . .    
301	G G G G A G G A . . .    
302	G G G G G G G G . . .    
303	A A A A A A A A . . .    
304	C C C C C C C C . . .    
305	C C C C C C C C . . .    
306	T T T T T T T T . . .    
307	G G G G G G G G . . .    
308	C C C C C C C C . . .    
309	C C C C C C C C . . .    
310	A A A A A A A A . . .    
311	G G G G G G G G . . .    
312	A A A A A A A A . . .    
313	A A A A A A A A . . .    
314	C C C C C C C C . . .    
315	T T T T T T T T . . .    
316	C C C C C C C C . . .    
317	A A A A A A A A . . .    
318	A A A A A A A A . . .    
319	A A A A A A A A . . .    
320	G G G G G G G G . . .    
321	C C C C C C C C . . .    
322	T T T T T T T T . . .    
323	A A A A A A A A . . .    
324	A A A A A A A A . . .    
325	A A A A A A A A . . .    
326	G G G G G G G G . . .    
327	A A A A A A A A . . .    
328	G G G G G G G G . . .    
329	A A A A A A A A . . .    
330	A A A A A A A A . . .    
331	T T T T T T T T . . .    
332	T T T T T T T T . . .    
333	T T T T T T T T . . .    
334	C C C C C C C C . . .    
335	A A A A A A A A . . .    
336	T T T T T T T T . . .    
337	C C C C C C C C . . .    
338	A A A A A A A A . . .    
339	T T T T T T T T . . .    
340	A A A A A A A A . . .    
341	G G G G G G G G . . .    
342	A A A A A A A A . . .    
343	A A A A A A A A . . .    
344	G G G G G G G G . . .    
345	A A A A A A A A . . .    
346	A A A A A A A A . . .    
347	A A A A A A A A . . .    
348	G G G G G G G G . . .    
349	A A A A A A A A . . .    
350	A A A A A A A A . . .    
351	G G G G G G G G . . .    
352	T T T T T T T T . . .    
353	T T T T T T T T . . .    
354	T T T T T T T T . . .    
355	T T T T T T T T . . .    
356	G G G G G G G G . . .    
357	G G G G G G G G . . .    
358	G G G G G G G G . . .    
359	C C C C C C C C . . .    
360	C C C C C C C C . . .    
361	C C C C C C C C . . .    
362	T T T T T T T T . . .    
363	G G G A G G G G . . .    
364	T T T T T T T T . . .    
365	G G G G G G G G . . .    
366	A A A A A A A A . . .    
367	A A A A A A A A . . .    
368	G G G G G G G G . . .    
369	A A A A A A A A . . .    
370	C C C C C C C C . . .    
371	C C C C C C C C . . .    
372	T T T T T T T T . . .    
373	G G G G G G G G . . .    
374	A A A A A A A A . . .    
375	A A A A A A A A . . .    
376	G G G G G G G G . . .    
377	T T T T T T T T . . .    
378	A A A A A A A A . . .    
379	T T T T T T T T . . .    
380	G G G G G G G G . . .    
381	G G G G G G G G . . .    
382	A A A A A A A A . . .    
383	A A A A A A A A . . .    
384	G G G G G G G G . . .    
385	A A A A A A A A . . .    
386	A A A A A A A A . . .    
387	T T T T T T T T . . .    
388	G G G G G G G G . . .    
389	T T T T T T T T . . .    
390	C C C C C C C C . . .    
391	A A A A A A A A . . .    
392	T T T T T T T T . . .    
393	T T T T T T T T . . .    
394	C C C C C C C C . . .    
395	A A A A A A A A . . .    
396	A A A A A A A A . . .    
397	G G G G G G G G . . .    
398	G G G G G G G G . . .    
399	G G G G G G G G . . .    
400	G G G G G G G G . . .    
401	T T T T T T T T . . .    
402	T T T T T T T T . . .    
403	C C C C C C C C . . .    
404	A A A A A A A A . . .    
405	A A A A A A A A . . .    
406	T T T T T T T T . . .    
407	A A A A A A A A . . .    
408	C C C C C C C C . . .    
409	A A A A A A A A . . .    
410	G G G G G G G G . . .    
411	A A A A A A A A . . .    
412	T T T T T T T T . . .    
413	G G G G G G G G . . .    
414	T T T T T T T T . . .    
415	A A A A A A A A . . .    
416	G G G G G G G G . . .    
417	A A A A A A A A . . .    
418	G G G G G G G G . . .    
419	A A A A A A A A . . .    
420	A A A A A A A A . . .    
421	A A A A A A A A . . .    
422	C C C C C C C C . . .    
423	T T T T T T T T . . .    
424	A A A A A A A A . . .    
425	A A A A A A A A . . .    
426	T T T T T T T T . . .    
427	G G G G G G G G . . .    
428	A A A A A A A A . . .    
429	T T T T T T T T . . .    
430	C C C C C C C C . . .    
431	G G G G G G G G . . .    
432	T T T T T T T T . . .    
433	G G G G G G G G . . .    
434	A A A A A A A A . . .    
435	T T T T T T T T . . .    
436	G G G G G G G G . . .    
437	A A A A A A A A . . .    
438	A A A A A A A A . . .    
439	T T T T T T T T . . .    
440	G G G G G G G G . . .    
441	C C C C C C C C . . .    
442	T T T T T T T T . . .    
443	C C C C C C C C . . .    
444	C C C C C C C C . . .    
445	A A A A A A A A . . .    
446	A A A A A A A A . . .    
447	A A A A A A A A . . .    
448	T T T T T T T T . . .    
449	G G G G G G G G . . .    
450	A A A A A A A A . . .    
451	G G G G G G G G . . .    
452	G G G G G G G G . . .    
453	A A A A A A A A . . .    
454	A A A A A A A A . . .    
455	G G G G G G G G . . .    
456	A A A A A A A A . . .    
457	G G G G G G G G . . .    
458	T T T T T T T T . . .    
459	T T T T T T T T . . .    
460	G G G G G G G G . . .    
461	G G G G G G G G . . .    
462	A A A A A A A A . . .    
463	A A A A A A A A . . .    
464	G G G G G G G G . . .    
465	A A A A A A A A . . .    
466	G G G G G G G G . . .    
467	G G G G G G G G . . .    
468	A A A A A A A A . . .    
469	G G G G G G G G . . .    
470	G G G G G G G G . . .    
471	A A A A A A A A . . .    
472	A A A A A A A A . . .    
473	A A A A A A A A . . .    
474	T T T T T T T T . . .    
475	T T T T T T T T . . .    
476	A A A A A A A A . . .    
477	G G G G G G G G . . .    
478	C C C C C C C C . . .    
479	A A A A A A A A . . .    
480	G G G G G G G G . . .    
481	A A A A A A A A . . .    
482	A A A A A A A A . . .    
483	T T T T T T T T . . .    
484	G G G G G G G G . . .    
485	G G G G G G G G . . .    
486	A A A A A A A A . . .    
487	G G G G G G G G . . .    
488	A A A A A A A A . . .    
489	C C C C C C C C . . .    
490	T T T T T T T T . . .    
491	G G G G G G G G . . .    
492	A A A A A A A A . . .    
493	T T T T T T T T . . .    
494	A A A A A A A A . . .    
495	T T T T T T T T . . .    
496	C C C C C C C C . . .    
497	A A A A A A A A . . .    
498	C C C C C C C C . . .    
499	A A A A A A A A . . .    
500	G G G G G G G G . . .    
501	A A A A A A A A . . .    
502	T T T T T T T T . . .    
503	G G G G G G G G . . .    
504	A A A A A A A A . . .    
505	A A A A A A A A . . .    
506	G G G G G G G G . . .    
507	A A A A A A A A . . .    
508	C C C C C C C C . . .    
509	A A A A A A A A . . .    
510	T T T T T T T T . . .    
511	G G G G G G G G . . .    
512	G G G G G G G G . . .    
513	C C C C C C C C . . .    
514	T T T T T T T T . . .    
515	A A A A A A A A . . .    
516	G G G G G G G G . . .    
517	A A A A A A A A . . .    
518	A A A A A A A A . . .    
519	G G G G G G G G . . .    
520	G G G G G G G G . . .    
521	T T T T T T T T . . .    
522	A A A A A A A A . . .    
523	C C C C C C C C . . .    
524	G G G G G G G G . . .    
525	A A A A A A A A . . .    
526	G G G G G G G G . . .    
527	A A G A A A A A . . .    
528	G G G G G G G G . . .    
529	C C C C C C C C . . .    
530	T T T T T T T T . . .    
531	T T T T T T T T . . .    
532	A A A A A A A A . . .    
533	G G G G G G G G . . .    
534	T T T T T T T T . . .    
535	G G G G G G G G . . .    
536	G G G G G G G G . . .    
537	G G G G G G G G . . .    
538	A A A A A A A A . . .    
539	A A A A A A A A . . .    
540	G G G G G G G G . . .    
541	C C C C C C C C . . .    
542	A A A T A A A A . . .    
543	T T T T T T T T . . .    
544	G G G G G G G G . . .    
545	A A A A A A A A . . .    
546	A A A A A A A A . . .    
547	G G G G G G G G . . .    
548	A A A A A A A A . . .    
549	A A A A A A A A . . .    
550	G G G G G G G G . . .    
551	A A A A A A A A . . .    
552	A A A A A A A A . . .    
553	G G G G G G G G . . .    
554	T T T T T T T T . . .    
555	T T T T T T T T . . .    
556	T T T T T T T T . . .    
557	G G G G G G G G . . .    
558	C C C C C C C C . . .    
559	T T T T T T T T . . .    
560	A A A A A A A A . . .    
561	A A A A A A A A . . .    
562	G G G G G G G G . . .    
563	A A A A A A A A . . .    
564	A A A A A A A A . . .    
565	G G G G G G G G . . .    
566	C C C C C C C C . . .    
567	G G G G G G G G . . .    
568	G G G G G G G G . . .    
569	G G G G G G G G . . .    
570	A A A A A A A A . . .    
571	G G G G G G G G . . .    
572	A A A A A A A A . . .    
573	G G G G G G G G . . .    
574	G G G G G G G G . . .    
575	T T T T T T T T . . .    
576	C C C C C C C C C . .    
577	T T T T T T T T T . .    
578	G G G G G G G G G . .    
579	C C C C C C C C C . .    
580	G G G G G G G G G . .    
581	A A A A A A A A A . .    
582	T T T T T T T T T . .    
583	T T T T T T T T T . .    
584	A A A A A A A A A . .    
585	A A A A A A A A A . .    
586	G G G G G G G G G . .    
587	G G G G G G G G G . .    
588	A A A A A A A A A . .    
589	T T T T T T T T T . .    
590	. G G G G G G G G . .    
591	. T T T T T T T T . .    
592	. T T T T T T T T . .    
593	. A A A A A A A A . .    
594	. A A A A A A A A . .    
595	. T T T T T T T T . .    
596	. C C C C C C C C . .    
597	. A A A A A A A A . .    
598	. A A A A A A A A . .    
599	. A A A A A A A A . .    
600	. A A A A A A A A . .    
601	. C C C C C C C C . .    
602	. G G G G G G G G . .    
603	. C C C C C C C C . .    
604	. A A A A A A A A . .    
605	. A A A A A A A A . .    
606	. T T T T T T T T . .    
607	. T T T T T T T T . .    
608	. G G G G G G G G . .    
609	. A A A A A A A A . .    
610	. G G G G G G G G . .    
611	. A A A A A A A A . .    
612	. C C C C C C C C . .    
613	. A A A A A A A A . .    
614	. C C C C C C C C . .    
615	. A A A A A A A A . .    
616	. A A A A A A A A . .    
617	. A A A A A A A A . .    
618	. C C C C C C C C . .    
619	. A A A A A A A A . .    
620	. A A A A A A A A . .    
621	. A A A A A A A A . .    
622	. A A A A A A A A . .    
623	. A A A A A A A A . .    
624	. A A A A A A A A . .    
625	. G G G G G G G G . .    
626	. G G G G G G G G . .    
627	. C C C C C C C C . .    
628	. T T T T T T T T . .    
629	. T T T T T T T T . .    
630	. T T T T T T T T . .    
631	. T T T T T T T T . .    
632	. T T T T T T T T . .    
633	. T T T T T T T T . .    
634	. T G G G G G G G G .    
635	. . A A A A A A A A A    
636	. . A A A A A A A A A    
637	. . G G G G G G G G G    
638	. . C C C C C C C C C    
639	. . C C C C C C C C C    
640	. . T T T T T T T T T    
641	. . C C C C C C C C C    
642	. . A A A A A A A A A    
643	. . G G G G G G G G G    
644	. . G G G G G G G G G    
645	. . A A A A A A A A A    
646	. . C C C C C C C C C    
647	. . T T T T T T T T T    
648	. . G G G G G G G G G    
649	. . A A A A A A A A A    
650	. . G G G G G G G G G    
651	. . G G G G G G G G G    
652	. . G G G G G G G G G    
653	. . C C C C C C C C C    
654	. . A A A A A A A A A    
655	. . T T T T T T T T T    
656	. . G G G G G G G G G    
657	. . C C C C C C C C C    
658	. . T T T T T T T T T    
659	. . T T T T T T T T T    
660	. . T T T T T T T T T    
661	. . T T T T T T T T T    
662	. . T T T T T T T T T    
663	. . A A A A A A A A A    
664	. . T T T T T T T T T    
665	. . T T T T T T T T T    
666	. . T T T T T T T T T    
667	. . A A A A A A A A A    
668	. . A A A A A A A A A    
669	. . A A A A A A A A A    
670	. . T T T T T T T T T    
671	. . G G G G G G G G G    
672	. . A A A A A A A A A    
673	. . A A A A A A A A A    
674	. . G G G G G G G G G    
675	. . T T T T T T T T T    
676	. . A A A A A A A A A    
677	. . T T T T T T T T T    
678	. . G G G G G G G G G    
679	. . T T T T T T T T T    
680	. . C C C C C C C C C    
681	. . A A A A A A A A A    
682	. . G G G G G G G G G    
683	. . G G G G G G G G G    
684	. . A A A A A A A A A    
685	. . C C C C C C C C C    
686	. . T T T T T T T T T    
687	. . G G G G G G G G G    
688	. . T T T T T T T T T    
689	. . A A A A A A A A A    
690	. . C C C C C C C C C    
691	. . A A A A A A A A A    
692	. . A A A A A A A A A    
693	. . T T T T T T T T T    
694	. . G G G G G G G G G    
695	. . T T T T T T T T T    
696	. . T T T T T T T T T    
697	. . T T T T T T T T T    
698	. . - - - - T - T T T  4	 indel  
699	. . - - - - T - T T T  4	 indel  
700	. . A A A A A A A A A    
701	. . T T T T T T T T T    
702	. . G G G G G G G G G    
703	. . T T . T T T T T T    
704	. . G G . G G G G G G    
705	. . C C . C C C C C C    
706	. . A A . A A A A A A    
707	. . A A . A A A A A A    
708	. . C C . C C C C C C    
709	. . . . . A A A A A A    
710	. . . . . A A A A A A    
711	. . . . . T T T T T T    
712	. . . . . T T T T T T    
713	. . . . . T T T T T T    
714	. . . . . T T T T T T    
715	. . . . . T T T T T T    
716	. . . . . A A A A A A    
717	. . . . . T T T T T T    
718	. . . . . T T T T T T    
719	. . . . . T T T T T T    
720	. . . . . T T T T T T    
721	. . . . . A A A A A A    
722	. . . . . A A A A A A    
723	. . . . . C C C C C C    
724	. . . . . T T T T T T    
725	. . . . . G G G G G G    
726	. . . . . C C C C C C    
727	. . . . . T T T T T T    
728	. . . . . G G G G G G    
729	. . . . . T T T T T T    
730	. . . . . T T T T T T    
731	. . . . . T T T T T T    
732	. . . . . T T T T T T    
733	. . . . . C C C C C C    
734	. . . . . A A A A A A    
735	. . . . . T T T T T T    
736	. . . . . G G G G G G    
737	. . . . . A A A A A A    
738	. . . . . A A A A A A    
739	. . . . . C C C C C C    
740	. . . . . C C C C C C    
741	. . . . . G G G G G G    
742	. . . . . T T T T T T    
743	. . . . . A A A A A A    
744	. . . . . G G G G G G    
745	. . . . . G G G G G G    
746	. . . . . T T T T T T    
747	. . . . . A A A A A A    
748	. . . . . A A A A A A    
749	. . . . . C C C C C C    
750	. . . . . T T T T T T    
751	. . . . . T T T T T T    
752	. . . . . C C C C C C    
753	. . . . . C C C C C C    
754	. . . . . A A A A A A    
755	. . . . . T T T T T T    
756	. . . . . A A A A A A    
757	. . . . . G G G G G G    
758	. . . . . A A A A A A    
759	. . . . . T T T T T T    
760	. . . . . G G G G G G    
761	. . . . . T T T T T T    
762	. . . . . T T T T T T    
763	. . . . . A A A A A A    
764	. . . . . C C C C C C    
765	. . . . . A A A A A A    
766	. . . . . G G G G G G    
767	. . . . . G G G G G G    
768	. . . . . C C C C C C    
769	. . . . . A A A G A A    
770	. . . . . T T T T T T    
771	. . . . . A A A A A A    
772	. . . . . T T T T T T    
773	. . . . . A A A A A A    
774	. . . . . T T T T T T    
775	. . . . . T T T T T T    
776	. . . . . T T T T T T    
777	. . . . . T T T T T T    
778	. . . . . A A A A A A    
779	. . . . . A A A A A A    
780	. . . . . A A A A A A    
781	. . . . . G G G G G G    
782	. . . . . T T T T T T    
783	. . . . . T T T T T T    
784	. . . . . G G G G G G    
785	. . . . . A A A A A A    
786	. . . . . A A A A A A    
787	. . . . . T T T T T T    
788	. . . . . G G G G G G    
789	. . . . . G G G G G G    
790	. . . . . A A A A A A    
791	. . . . . T T T T T T    
792	. . . . . G G G G G G    
793	. . . . . T T T T T T    
794	. . . . . T T T T T T    
795	. . . . . A A A A A A    
796	. . . . . T T T T T T    
797	. . . . . A A A A A A    
798	. . . . . T T T T T T    
799	. . . . . A A A A A A    
800	. . . . . T T T T T T    
801	. . . . . C C C C C C    
802	. . . . . C C C C C C    
803	. . . . . T T T T T T    
804	. . . . . C C C C C C    
805	. . . . . T T T T T T    
806	. . . . . T T T T T T    
807	. . . . . T T T T T T    
808	. . . . . T T T T T T    
809	. . . . . T T T T T T    
810	. . . . . A A A A A A    
811	. . . . . A A A A A A    
812	. . . . . A A A A A A    
813	. . . . . A A A A A A    
814	. . . . . G G G G G G    
815	. . . . . T T T T T T    
816	. . . . . C C C C C C    
817	. . . . . T T T T T T    
818	. . . . . A A A A A A    
819	. . . . . T T T T T T    
820	. . . . . T T T T T T    
821	. . . . . T T T T T T    
822	. . . . . C C C C C C    
823	. . . . . T T T T T T    
824	. . . . . T T T T T T    
825	. . . . . C C C C C C    
826	. . . . . A A A A A A    
827	. . . . . A A A A A A    
828	. . . . . G G G G G G    
829	. . . . . A A A A A A    
830	. . . . . T T T T T T    
831	. . . . . G G G G G G    
832	. . . . . C C C C C C    
833	. . . . . A A A A A A    
834	. . . . . T T T T T T    
835	. . . . . T T T T T T    
836	. . . . . G G G G G G    
837	. . . . . A A A A A A    
838	. . . . . G G G G G G    
839	. . . . . G G G G G G    
840	. . . . . G G G G G G    
841	. . . . . T T T T T T    
842	. . . . . T T T T T T    
843	. . . . . A A A A A A    
844	. . . . . T T T T T T    
845	. . . . . G G G G G G    
846	. . . . . A A A A A A    
847	. . . . . A A A A A A    
848	. . . . . C C C C C C    
849	. . . . . A A A A A A    
850	. . . . . C C C C C C    
851	. . . . . A A A A A A    
852	. . . . . G G G G G G    
853	. . . . . G G G G G G    
854	. . . . . A A A A A A    
855	. . . . . A A A A A A    
856	. . . . . A A A A A A    
857	. . . . . T T T T T T    
858	. . . . . G G G G G G    
859	. . . . . A A A A A A    
860	. . . . . A A A A A A    
861	. . . . . T T T T T T    
862	. . . . . C C C C C C    
863	. . . . . C C C C C C    
864	. . . . . C C C C C C    
865	. . . . . A A A A A A    
866	. . . . . C C C C C C    
867	. . . . . A A A A A A    
868	. . . . . T T T T T T    
869	. . . . . C C C C C C    
870	. . . . . C C C C C C    
871	. . . . . T T T T T T    
872	. . . . . T T T T T T    
873	. . . . . G G G G G G    
874	. . . . . T T T T T T    
875	. . . . . A A A A A A    
876	. . . . . C C C C C C    
877	. . . . . C C C C C C    
878	. . . . . A A A A A A    
879	. . . . . T T T T T T    
880	. . . . . G G G G G G    
881	. . . . . G G G G G G    
882	. . . . . A A A A A A    
883	. . . . . C C C C C C    
884	. . . . . A A A A A A    
885	. . . . . A A A A A A    
886	. . . . . G G G G G G    
887	. . . . . G G G G G G    
888	. . . . . G G G G G G    
889	. . . . . T T T T T T    
890	. . . . . A A A A A A    
891	. . . . . T T T T T T    
892	. . . . . A A A A A A    
893	. . . . . G G G G G G    
894	. . . . . T T T T T T    
895	. . . . . T T T T T T    
896	. . . . . T T T T T T    
897	. . . . . A A A A A A    
898	. . . . . A A A A A A    
899	. . . . . T T T T T T    
900	. . . . . T T T T T T    
901	. . . . . A A A A A A    
902	. . . . . A A A A A A    
903	. . . . . A A A A A A    
904	. . . . . A A A A A A    
905	. . . . . C C C C C C    
906	. . . . . T T T T T T    
907	. . . . . G G G G G G    
908	. . . . . T T T T T T    
909	. . . . . T T T T T T    
910	. . . . . A A A A A A    
911	. . . . . C C C C C C    
912	. . . . . C C C C C C    
913	. . . . . C C C C C C    
914	. . . . . A . . A A A    
915	. . . . . A . . A A A    
916	. . . . . T . . T T T    
917	. . . . . G . . G G G    
918	. . . . . C . . T T T    
919	. . . . . . . . G G G    
920	. . . . . . . . A G G    
921	. . . . . . . . . T T    
922	. . . . . . . . . G G    
923	. . . . . . . . . G G    
924	. . . . . . . . . C C    
925	. . . . . . . . . T T    
926	. . . . . . . . . T T    
927	. . . . . . . . . G G    
928	. . . . . . . . . T T    
929	. . . . . . . . . C C    
930	. . . . . . . . . T T    
931	. . . . . . . . . T T    
932	. . . . . . . . . C C    
933	. . . . . . . . . T T    
934	. . . . . . . . . G G    
935	. . . . . . . . . T T    
936	. . . . . . . . . G G    
937	. . . . . . . . . A A    
938	. . . . . . . . . G G    
939	. . . . . . . . . A A    
940	. . . . . . . . . A A    
941	. . . . . . . . . A A    
942	. . . . . . . . . T T    
943	. . . . . . . . . T T    
944	. . . . . . . . . G G    
945	. . . . . . . . . A A    
946	. . . . . . . . . C C    
947	. . . . . . . . . A A    
948	. . . . . . . . . A A    
949	. . . . . . . . . A A    
950	. . . . . . . . . A A    
951	. . . . . . . . . T T    
952	. . . . . . . . . A A    
953	. . . . . . . . . T T    
954	. . . . . . . . . A A    
955	. . . . . . . . . C C    
956	. . . . . . . . . T T    
957	. . . . . . . . . A A    
958	. . . . . . . . . A A    
959	. . . . . . . . . A A    
960	. . . . . . . . . T T    
961	. . . . . . . . . T T    
962	. . . . . . . . . C C    
963	. . . . . . . . . A A    
964	. . . . . . . . . A A    
965	. . . . . . . . . A A    
966	. . . . . . . . . C C    
967	. . . . . . . . . A A    
968	. . . . . . . . . A A    
969	. . . . . . . . . T T    
970	. . . . . . . . . A A    
971	. . . . . . . . . A A    
972	. . . . . . . . . C C    
973	. . . . . . . . . A A    
974	. . . . . . . . . A A    
975	. . . . . . . . . G G    
976	. . . . . . . . . G G    
977	. . . . . . . . . G G    
978	. . . . . . . . . T T    
979	. . . . . . . . . T T    
980	. . . . . . . . . T T    
981	. . . . . . . . . T T    
982	. . . . . . . . . C C    
983	. . . . . . . . . A A    
984	. . . . . . . . . A A    
985	. . . . . . . . . T T    
986	. . . . . . . . . A A    
987	. . . . . . . . . G G    
988	. . . . . . . . . C C    
989	. . . . . . . . . C C    
990	. . . . . . . . . T T    
991	. . . . . . . . . T T    
992	. . . . . . . . . G G    
993	. . . . . . . . . C C    
994	. . . . . . . . . T T    
995	. . . . . . . . . T T    
996	. . . . . . . . . A A    
997	. . . . . . . . . T T    
998	. . . . . . . . . G G    
999	. . . . . . . . . A A    
1000	. . . . . . . . . A A    
1001	. . . . . . . . . G G    
1002	. . . . . . . . . C C    
1003	. . . . . . . . . A A    
1004	. . . . . . . . . G G    
1005	. . . . . . . . . C C    
1006	. . . . . . . . . C C    
1007	. . . . . . . . . A A    
1008	. . . . . . . . . A A    
1009	. . . . . . . . . A A    
1010	. . . . . . . . . A A    
1011	. . . . . . . . . A A    
1012	. . . . . . . . . A A    
1013	. . . . . . . . . G G    
1014	. . . . . . . . . T T    
1015	. . . . . . . . . A A    
1016	. . . . . . . . . C C    
1017	. . . . . . . . . A A    
1018	. . . . . . . . . A A    
1019	. . . . . . . . . A A    
1020	. . . . . . . . . G G    
1021	. . . . . . . . . C C    
1022	. . . . . . . . . C C    
1023	. . . . . . . . . A A    
1024	. . . . . . . . . A A    
1025	. . . . . . . . . C C    
1026	. . . . . . . . . A A    
1027	. . . . . . . . . G G    
1028	. . . . . . . . . T T    
1029	. . . . . . . . . C C    
1030	. . . . . . . . . A A    
1031	. . . . . . . . . C C    
1032	. . . . . . . . . A A    
1033	. . . . . . . . . T T    
1034	. . . . . . . . . T T    
1035	. . . . . . . . . T T    
1036	. . . . . . . . . C C    
1037	. . . . . . . . . A A    
1038	. . . . . . . . . A A    
1039	. . . . . . . . . T T    
1040	. . . . . . . . . G G    
1041	. . . . . . . . . C C    
1042	. . . . . . . . . C C    
1043	. . . . . . . . . A A    
1044	. . . . . . . . . T T    
1045	. . . . . . . . . C C    
1046	. . . . . . . . . C C    
1047	. . . . . . . . . A A    
1048	. . . . . . . . . A A    
1049	. . . . . . . . . G G    
1050	. . . . . . . . . C C    
1051	. . . . . . . . . A A    
1052	. . . . . . . . . T T    
1053	. . . . . . . . . G G    
1054	. . . . . . . . . A A    
1055	. . . . . . . . . G G    
1056	. . . . . . . . . G G    
1057	. . . . . . . . . G G    
1058	. . . . . . . . . A A    
1059	. . . . . . . . . A A    
1060	. . . . . . . . . G G    
1061	. . . . . . . . . A A    
1062	. . . . . . . . . T T    
1063	. . . . . . . . . G G    
1064	. . . . . . . . . G G    
1065	. . . . . . . . . A A    
1066	. . . . . . . . . G G    
1067	. . . . . . . . . A A    
1068	. . . . . . . . . G G    
1069	. . . . . . . . . A A    
1070	. . . . . . . . . T T    
1071	. . . . . . . . . A A    
1072	. . . . . . . . . C C    
1073	. . . . . . . . . T T    
1074	. . . . . . . . . A A    
1075	. . . . . . . . . A A    
1076	. . . . . . . . . T T    
1077	. . . . . . . . . C C    
1078	. . . . . . . . . T T    
1079	. . . . . . . . . A A    
1080	. . . . . . . . . T T    
1081	. . . . . . . . . G G    
1082	. . . . . . . . . G G    
1083	. . . . . . . . . A A    
1084	. . . . . . . . . A A    
1085	. . . . . . . . . G G    
1086	. . . . . . . . . C C    
1087	. . . . . . . . . A A    
1088	. . . . . . . . . T T    
1089	. . . . . . . . . T T    
1090	. . . . . . . . . C C    
1091	. . . . . . . . . G G    
1092	. . . . . . . . . C C    
1093	. . . . . . . . . G G    
1094	. . . . . . . . . T T    
1095	. . . . . . . . . A A    
1096	. . . . . . . . . A A    
1097	. . . . . . . . . A A    
1098	. . . . . . . . . C C    
1099	. . . . . . . . . A A    
1100	. . . . . . . . . A A    
1101	. . . . . . . . . C C    
1102	. . . . . . . . . C C    
1103	. . . . . . . . . T T    
1104	. . . . . . . . . T T    
1105	. . . . . . . . . G G    
1106	. . . . . . . . . G G    
1107	. . . . . . . . . G G    
1108	. . . . . . . . . A A    
1109	. . . . . . . . . A A    
1110	. . . . . . . . . A A    
1111	. . . . . . . . . G G    
1112	. . . . . . . . . A A    
1113	. . . . . . . . . A A    
1114	. . . . . . . . . T T    
1115	. . . . . . . . . A A    
1116	. . . . . . . . . G G    
1117	. . . . . . . . . C C    
1118	. . . . . . . . . C C    
1119	. . . . . . . . . C C    
1120	. . . . . . . . . G G    
1121	. . . . . . . . . A A    
1122	. . . . . . . . . C C    
1123	. . . . . . . . . G G    
1124	. . . . . . . . . T T    
1125	. . . . . . . . . C C    
1126	. . . . . . . . . A A    
1127	. . . . . . . . . C C    
1128	. . . . . . . . . A A    
1129	. . . . . . . . . T T    
1130	. . . . . . . . . A A    
1131	. . . . . . . . . T T    
1132	. . . . . . . . . C C    
1133	. . . . . . . . . A A    
1134	. . . . . . . . . G G    
1135	. . . . . . . . . T T    
1136	. . . . . . . . . T T    
1137	. . . . . . . . . A A    
1138	. . . . . . . . . C C    
1139	. . . . . . . . . C C    
1140	. . . . . . . . . A A    
1141	. . . . . . . . . A A    
1142	. . . . . . . . . G G    
1143	. . . . . . . . . T T    
1144	. . . . . . . . . C C    
1145	. . . . . . . . . T T    
1146	. . . . . . . . . T T    
1147	. . . . . . . . . C C    
1148	. . . . . . . . . G G    
1149	. . . . . . . . . A A    
1150	. . . . . . . . . G G    
1151	. . . . . . . . . C C    
1152	. . . . . . . . . C C    
1153	. . . . . . . . . T T    
1154	. . . . . . . . . C C    
1155	. . . . . . . . . C C    
1156	. . . . . . . . . T T    
1157	. . . . . . . . . G G    
1158	. . . . . . . . . C C    
1159	. . . . . . . . . C C    
1160	. . . . . . . . . T T    
1161	. . . . . . . . . G G    
1162	. . . . . . . . . G G    
1163	. . . . . . . . . A A    
1164	. . . . . . . . . G G    
1165	. . . . . . . . . C C    
1166	. . . . . . . . . C C    
1167	. . . . . . . . . C C    
1168	. . . . . . . . . A A    
1169	. . . . . . . . . G G    
1170	. . . . . . . . . C C    
1171	. . . . . . . . . T T    
1172	. . . . . . . . . G G    
1173	. . . . . . . . . G G    
1174	. . . . . . . . . C C    
1175	. . . . . . . . . A A    
1176	. . . . . . . . . G G    
1177	. . . . . . . . . A A    
1178	. . . . . . . . . C C    
1179	. . . . . . . . . T T    
1180	. . . . . . . . . G G    
1181	. . . . . . . . . C C    
1182	. . . . . . . . . T T    
1183	. . . . . . . . . A A    
1184	. . . . . . . . . T T    
1185	. . . . . . . . . T T    
1186	. . . . . . . . . G G    
1187	. . . . . . . . . A A    
1188	. . . . . . . . . A A    
1189	. . . . . . . . . C C    
1190	. . . . . . . . . T T    
1191	. . . . . . . . . G G    
1192	. . . . . . . . . A A    
1193	. . . . . . . . . A A    
1194	. . . . . . . . . A A    
1195	. . . . . . . . . C C    
1196	. . . . . . . . . A A    
1197	. . . . . . . . . G G    
1198	. . . . . . . . . A A    
1199	. . . . . . . . . C C    
1200	. . . . . . . . . T T    
1201	. . . . . . . . . T T    
1202	. . . . . . . . . G G    
1203	. . . . . . . . . T T    
1204	. . . . . . . . . A A    
1205	. . . . . . . . . A A    
1206	. . . . . . . . . T T    
1207	. . . . . . . . . A A    
1208	. . . . . . . . . A A    
1209	. . . . . . . . . T T    
1210	. . . . . . . . . G G    
1211	. . . . . . . . . A A    
1212	. . . . . . . . . T T    
1213	. . . . . . . . . G G    
1214	. . . . . . . . . T T    
1215	. . . . . . . . . C C    
1216	. . . . . . . . . A A    
1217	. . . . . . . . . T T    
1218	. . . . . . . . . A A    
1219	. . . . . . . . . G G    
1220	. . . . . . . . . A A    
1221	. . . . . . . . . A A    
1222	. . . . . . . . . C C    
1223	. . . . . . . . . C C    
1224	. . . . . . . . . G G    
1225	. . . . . . . . . T T    
1226	. . . . . . . . . G G    
1227	. . . . . . . . . T T    
1228	. . . . . . . . . G G    
1229	. . . . . . . . . G G    
1230	. . . . . . . . . G G    
1231	. . . . . . . . . A A    
1232	. . . . . . . . . C C    
1233	. . . . . . . . . A A    
1234	. . . . . . . . . A A    
1235	. . . . . . . . . A A    
1236	. . . . . . . . . G G    
1237	. . . . . . . . . G G    
1238	. . . . . . . . . C C    
1239	. . . . . . . . . G G    
1240	. . . . . . . . . G G    
1241	. . . . . . . . . T T    
1242	. . . . . . . . . G G    
1243	. . . . . . . . . G G    
1244	. . . . . . . . . A A    
1245	. . . . . . . . . G G    
1246	. . . . . . . . . G G    
1247	. . . . . . . . . C C    
1248	. . . . . . . . . A A    
1249	. . . . . . . . . G G    
1250	. . . . . . . . . G G    
1251	. . . . . . . . . G G    
1252	. . . . . . . . . A A    
1253	. . . . . . . . . A A    
1254	. . . . . . . . . T T    
1255	. . . . . . . . . G G    
1256	. . . . . . . . . A A    
1257	. . . . . . . . . G G    
1258	. . . . . . . . . C C    
1259	. . . . . . . . . C C    
1260	. . . . . . . . . A A    
1261	. . . . . . . . . G G    
1262	. . . . . . . . . G G    
1263	. . . . . . . . . C C    
1264	. . . . . . . . . A A    
1265	. . . . . . . . . G G    
1266	. . . . . . . . . A A    
1267	. . . . . . . . . C C    
1268	. . . . . . . . . T T    
1269	. . . . . . . . . G G    
1270	. . . . . . . . . G G    
1271	. . . . . . . . . C C    
1272	. . . . . . . . . C C    
1273	. . . . . . . . . A A    
1274	. . . . . . . . . G G    
1275	. . . . . . . . . G G    
1276	. . . . . . . . . G G    
1277	. . . . . . . . . G G    
1278	. . . . . . . . . A A    
1279	. . . . . . . . . G G    
1280	. . . . . . . . . . T    
1281	. . . . . . . . . . A    
1282	. . . . . . . . . . G    
1283	. . . . . . . . . . A    
1284	. . . . . . . . . . C    
1285	. . . . . . . . . . G    
1286	. . . . . . . . . . G    
1287	. . . . . . . . . . G    
1288	. . . . . . . . . . T    
1289	. . . . . . . . . . C    
1290	. . . . . . . . . . T    
1291	. . . . . . . . . . G    
1292	. . . . . . . . . . G    
1293	. . . . . . . . . . G    
1294	. . . . . . . . . . G    
1295	. . . . . . . . . . G    
1296	. . . . . . . . . . C    
1297	. . . . . . . . . . C    
1298	. . . . . . . . . . A    
1299	. . . . . . . . . . C    
1300	. . . . . . . . . . T    
1301	. . . . . . . . . . G    
1302	. . . . . . . . . . A    
1303	. . . . . . . . . . C    
1304	. . . . . . . . . . A    
1305	. . . . . . . . . . T    
1306	. . . . . . . . . . C    
1307	. . . . . . . . . . A    
1308	. . . . . . . . . . G    
1309	. . . . . . . . . . G    
1310	. . . . . . . . . . G    
1311	. . . . . . . . . . C    
1312	. . . . . . . . . . G    
1313	. . . . . . . . . . T    
1314	. . . . . . . . . . G    
1315	. . . . . . . . . . G    
1316	. . . . . . . . . . C    
1317	. . . . . . . . . . A    
1318	. . . . . . . . . . C    
1319	. . . . . . . . . . A    
1320	. . . . . . . . . . T    
1321	. . . . . . . . . . G    
1322	. . . . . . . . . . T    
1323	. . . . . . . . . . C    
1324	. . . . . . . . . . A    
1325	. . . . . . . . . . T    
1326	. . . . . . . . . . C    
1327	. . . . . . . . . . T    
1328	. . . . . . . . . . A    
1329	. . . . . . . . . . G    
1330	. . . . . . . . . . C    
1331	. . . . . . . . . . A    
1332	. . . . . . . . . . C    
1333	. . . . . . . . . . A    
1334	. . . . . . . . . . G    
1335	. . . . . . . . . . C    
1336	. . . . . . . . . . T    
1337	. . . . . . . . . . T    
1338	. . . . . . . . . . G    
1339	. . . . . . . . . . T    
1340	. . . . . . . . . . A    
1341	. . . . . . . . . . A    
1342	. . . . . . . . . . T    
1343	. . . . . . . . . . C    
1344	. . . . . . . . . . A    
1345	. . . . . . . . . . G    
1346	. . . . . . . . . . C    
1347	. . . . . . . . . . A    
1348	. . . . . . . . . . C    
1349	. . . . . . . . . . A    
1350	. . . . . . . . . . G    
1351	. . . . . . . . . . C    
1352	. . . . . . . . . . T    
1353	. . . . . . . . . . C    
1354	. . . . . . . . . . T    
1355	. . . . . . . . . . T    
1356	. . . . . . . . . . C    
1357	. . . . . . . . . . C    
1358	. . . . . . . . . . A    
1359	. . . . . . . . . . T    
1360	. . . . . . . . . . G    
1361	. . . . . . . . . . C    
1362	. . . . . . . . . . A    
1363	. . . . . . . . . . T    
1364	. . . . . . . . . . C    
1365	. . . . . . . . . . G    
1366	. . . . . . . . . . T    
1367	. . . . . . . . . . C    
1368	. . . . . . . . . . A    
1369	. . . . . . . . . . G    
1370	. . . . . . . . . . C    
1371	. . . . . . . . . . A    
1372	. . . . . . . . . . T    
1373	. . . . . . . . . . G    
1374	. . . . . . . . . . T    
1375	. . . . . . . . . . C    
1376	. . . . . . . . . . C    
1377	. . . . . . . . . . A    
1378	. . . . . . . . . . C    
1379	. . . . . . . . . . T    
1380	. . . . . . . . . . C    
1381	. . . . . . . . . . G    
1382	. . . . . . . . . . T    
1383	. . . . . . . . . . C    
1384	. . . . . . . . . . T    
1385	. . . . . . . . . . G    
1386	. . . . . . . . . . C    
1387	. . . . . . . . . . C    
1388	. . . . . . . . . . C    
1389	. . . . . . . . . . A    
1390	. . . . . . . . . . G    
1391	. . . . . . . . . . A    
1392	. . . . . . . . . . A    
1393	. . . . . . . . . . C    
1394	. . . . . . . . . . C    
1395	. . . . . . . . . . A    
1396	. . . . . . . . . . C    
1397	. . . . . . . . . . A    
1398	. . . . . . . . . . T    
1399	. . . . . . . . . . A    
1400	. . . . . . . . . . G    
1401	. . . . . . . . . . C    
1402	. . . . . . . . . . A    
1403	. . . . . . . . . . C    
1404	. . . . . . . . . . A    
1405	. . . . . . . . . . A    
1406	. . . . . . . . . . C    
1407	. . . . . . . . . . C    
1408	. . . . . . . . . . A    
1409	. . . . . . . . . . G    
1410	. . . . . . . . . . A    
1411	. . . . . . . . . . G    
1412	. . . . . . . . . . G    
1413	. . . . . . . . . . C    
1414	. . . . . . . . . . T    
1415	. . . . . . . . . . G    
1416	. . . . . . . . . . C    
1417	. . . . . . . . . . A    
1418	. . . . . . . . . . G    
1419	. . . . . . . . . . G    
1420	. . . . . . . . . . T    
1421	. . . . . . . . . . C    
1422	. . . . . . . . . . A    
1423	. . . . . . . . . . G    
1424	. . . . . . . . . . A    
1425	. . . . . . . . . . A    
1426	. . . . . . . . . . A    
1427	. . . . . . . . . . G    
1428	. . . . . . . . . . A    
1429	. . . . . . . . . . G    
1430	. . . . . . . . . . C    
1431	. . . . . . . . . . A    
1432	. . . . . . . . . . G    
1433	. . . . . . . . . . A    
1434	. . . . . . . . . . C    
1435	. . . . . . . . . . T    
1436	. . . . . . . . . . G    
1437	. . . . . . . . . . C    
1438	. . . . . . . . . . C    
1439	. . . . . . . . . . G    
1440	. . . . . . . . . . T    
1441	. . . . . . . . . . A    
1442	. . . . . . . . . . G    
1443	. . . . . . . . . . C    
1444	. . . . . . . . . . C    
1445	. . . . . . . . . . T    
1446	. . . . . . . . . . G    
1447	. . . . . . . . . . G    
1448	. . . . . . . . . . A    
1449	. . . . . . . . . . A    
1450	. . . . . . . . . . G    
1451	. . . . . . . . . . G    
1452	. . . . . . . . . . G    
1453	. . . . . . . . . . C    
1454	. . . . . . . . . . C    
1455	. . . . . . . . . . A    
1456	. . . . . . . . . . A    
1457	. . . . . . . . . . A    
1458	. . . . . . . . . . A    
1459	. . . . . . . . . . A    
1460	. . . . . . . . . . G    
1461	. . . . . . . . . . C    
1462	. . . . . . . . . . A    
1463	. . . . . . . . . . C    
1464	. . . . . . . . . . C    
1465	. . . . . . . . . . C    
1466	. . . . . . . . . . C    
1467	. . . . . . . . . . C    
1468	. . . . . . . . . . C
```
